# Supplementary material for: Nutritional inadequacies in commercial vegan foods for dogs and cats
Source: PLoS One. 2020 Jan 17;15(1):e0227046. doi: 10.1371/journal.pone.0227046 (PMC6968870; doi:10.1371/journal.pone.0227046)
Supplement: Attachment — Submitted filename: PONE-D-19-28038.pdf [file pone.0227046.s001.pdf]

Manuscript

Click here to download Manuscript Zafalon et al. 2019 PLOS ONE 07-10-2019[23256].docx

Nutritional inadequacies in commercial vegan foods for dogs and cats

Rafael Vessecchi Amorim Zafalon[1], Larissa Wünsche Risolia[1], Thiago Henrique Annibale Vendramini[1], Roberta Bueno Ayres Rodrigues[1], Vivian Pedrinelli[1], Fabio Alves Teixeira[1], Mariana Fragoso Rentas[1], Marcio Antonio Brunetto[1]

[1]School of Veterinary Medicine and Animal Science, University of São Paulo. São Paulo/Pirassununga, Brazil

*Corresponding author

E-mail: mabrunetto@usp.br (MAB)

Abstract

The objective of this study was to evaluate the macronutrients composition, fatty acid and amino acid profiles, and essential minerals content of vegan foods for dogs and cats available in the Brazilian market and to compare the results with FEDIAF (2018) recommendations. Four vegan pet foods are available (three for dogs and one for cats), and in the present study we evaluated all of them. The comparisons were made in a descriptive way. Arachidonic acid was not detected in any food. Regarding the FEDIAF recommendations, one food for dogs had low calcium, another had low potassium and a third had low sodium. The cat food presented potassium content lower than recommended. The Ca:P ratio did not meet the minimum recommendation in any of the foods analyzed. Copper concentrations exceeded the legal limit in all foods. Zinc concentrations exceeded this limit in two foods (one for dogs and one for cats). One of the dog foods did not meet the minimum recommendation for methionine and the cat food did not meet the minimum recommendation for arginine. All foods met the minimum recommendations for macronutrients. In addition, when the amount of nutrients consumed by animals with low energy requirements was simulated, in addition to the same non-conformities described above, it was observed that the cat food does not meet the minimum recommended of protein and taurine in unit/$Kg^{0.67}$. It was concluded that all foods analyzed had one or more nutrients below the recommended levels and some presented zinc and copper excess, therefore, these foods should not be recommended for dogs and cats, although they are available in the Brazilian market, because dietary deficiencies found may lead to health risks for dogs and cats. Furthermore, manufacturers should review their formulations to ensure the nutritional adequacy of these foods.

Introduction

Veganism is considered a stricter form of vegetarianism and involves practices other than dietary beliefs. Adepts of this movement exclude meat, seafood, eggs, dairy products, and all other animal products from their eating habits. Among the reasons that lead to the adoption of this lifestyle are health concerns, sustainability/environmental preservation, empathy for animals and ethical concerns [1].

According to a previous survey, about 14% of Brazilians declare themselves as vegetarian or vegan and this number seems to be growing [2]. The ethical appeal of veganism is also reflected in the search for food that is free of ingredients of animal origin for their pets, which can have troubling implications for animals, especially cats.

Considering the physiology of dogs and cats, which classifies them respectively as optional and strict carnivores, the adequacy of these diets in supplying the minimum requirement of essential nutrients has been questioned. The proper formulation of this type of food is a challenge since several of these nutrients are found mainly in ingredients of animal origin. A previous study found that only two out of 12 commercial vegetarian foods analyzed met the recommendations of the species for which they were intended [3].

Increasing adherents to veganism concurrently stimulate the development of nutritionally complete and balanced plant-based diets to ensure the nutritional requirements of these animals, while simultaneously meeting the ethical needs of owners. Almost half of the owners interested in providing plant-based diets want more information to ensure the nutritional adequacy of these foods [1], which demonstrates their concern about pet food quality. However, few studies have evaluated the composition of vegan foods or the impact on health of dogs and cats fed with this type of food.

Considering the above-mentioned facts, the objective of this study was to evaluate the macronutrient composition, fatty acid and amino acids profile, and essential minerals content

of vegan foods ~~for dogs and cats~~ available in the Brazilian market, in order to verify the compliance of these nutrients with the recommendations for these species.

Material and methods

In the Brazilian market, four vegan pet foods are commercialized (three for dogs and one for cats), all dried and extruded. In the present study, these four foods were evaluated. The nutritional composition of these foods is described in Table 1.

Table 1. Ingredient composition declared on the label of the vegan pet foods analyzed in this study.

Foods	Ingredients
**Food A**	Organic whole wheat flour, organic soybean meal, organic soybean oil, yeast extract, brewer's yeast, sugar cane fiber, chickpea, organic brown rice flour, sugar cane fiber, wheat gluten, organic flaxseed, organic chia, dehydrated carrot, olive oil, dicalcium phosphate, bentonite, vitamins (A, D, E, K, C, B1, B2, B12, biotin, calcium pantothenate, folic acid, niacin, choline chloride), minerals (iron sulfate, copper sulfate, calcium iodate, potassium chloride, zinc oxide, manganese oxide, sodium selenite), methionine, plant extracts (yucca, rosemary, green tea and mint) (0.06 %).
**Food B**	Broken rice, whole corn, corn gluten meal, soy bran, wheat bran, hydrolyzed yeast, yeast cell wall, yucca extract (0.025%), flax flour, probiotic additive (0.01%), prebiotic additive (0.02%), beet pulp, sodium chloride, degummed soybean oil (7.5%), hydrogenated soybean oil, folic acid, pantothenic acid, nicotinic acid, biotin, choline chloride, calcium iodate, pyridoxine, riboflavin, sodium selenite, cobalt sulphate, copper sulphate, dicalcium phosphate, calcium carbonate, manganese sulphate, magnesium sulphate, zinc sulphate, ferrous sulphate, vitamin A, vitamin B12, vitamin D2, vitamin E, vitamin K, l-lysine, dl-methionine, natural dye of urucum (0.06%), bentonite, dolomite, algae flour, potassium sorbate, propionic acid and natural antioxidant (0.05%) (tocopherol, silicon dioxide, rosemary oil, citric acid).
**Food C**	Ground whole maize, wheat bran, soybean meal, broken rice, corn gluten meal-60, brewer's dry yeast, yeast extract, degummed soybean oil (7%: omega 3 0 - .055% and omega 6 - 3.64%), calcitic limestone, dicalcium phosphate, sodium chloride (common salt), potassium chloride, vehicle q.s.p, natural antioxidant (0.06% - vegetable oil, citric acid, silicon dioxide, tocopherol extract, rosemary essence), yucca extract (0.0625%), propionic acid, calcium sodium aluminosilicate, potassium sorbate, flavour additive (dried sugar cane yeast, dried brewer's yeast, dextrose,

	phosphoric acid, potassium sorbate, tocopherol, rosemary oil), l-lysine, dl-methionine, zinc oxide, zinc amino acid chelate, copper sulfate, iron sulfate, manganese monoxide, calcium iodate, sodium selenite, selenium organic, choline chloride, folic acid, nicotinic acid, biotin, calcium pantothenate, vitamin A, vitamin B1, vitamin B12, vitamin B6, vitamin D3, vitamin E, vitamin K3, sodium hexametaphosphate, mannanoligosaccharide (0.2%), bacillus subtilis (0.038%).
**Food D**	Ground whole maize corn gluten meal, soy bran, broken rice, pea protein, wheat bran, hydrolyzed yeast, yeast cell wall, yucca extract (0.025%), flax flour, (0.5%), probiotic additive (0.01%), prebiotic additive (0.02%), beet pulp, sodium chloride, dehydrated soybean oil (7.5%), hydrogenated soybean oil, folic acid, pantothenic acid, nicotinic acid, biotin, choline chloride, calcium iodate, pyridoxine, riboflavin, vitamin b1, sodium selenite, cobalt sulphate, copper sulphate, dicalcium phosphate, calcium carbonate, manganese sulphate, magnesium sulphate, zinc sulfate, ferrous sulphate, vitamin A, vitamin B12, vitamin D2, vitamin E, vitamin K, l-lysine, dl-methionine, l-carnitine, taurine (0.15%), natural dye of urucum %, bentonite, dolomite, algae flour, potassium sorbate, phosphoric acid, citric acid, propionic acid and natural antioxidant (0.05%) (tocopherol, silicon dioxide, rosemary oil, citric acid).

normally ingredients listed by propn of ingoing weight – is this true here?

Bromatological analyzes

The four foods were previously ground in a hammer mill equipped with a 1mm sieve. The dry matter was determined in an oven at 105°C (Fanen 315, São Paulo, Brazil) for eight hours according to AOAC [4]. Crude protein (CP) analysis was performed by the Kjeldahl method [4]. The ethereal extract was performed by the Soxhlet method after acid hydrolysis, and ash was determined by muffle incineration at 550°C [4]. The crude fiber was determined by the Weende method [5]. The analyses were performed in duplicate, and repetition was performed when the coefficient of variation was greater than 5.0%.

Determination of fatty acid profile

The fatty acid profile of the foods was determined by gas chromatography, according to ISO [6], in the Laboratory of Biochemistry of Microorganisms and Plants, Department of Technology of the School of Agrarian and Veterinary Sciences of the São Paulo State University "Júlio de Mesquita Filho", Jaboticabal, Brazil.

Determination of minerals

For mineral analyses the samples were prepared by the wet method. A 200mg sample was conditioned in a 100mL test tube in which 4mL of concentrated nitric acid (HNO_3) was added. After this process, the tubes rested for 30 minutes. The samples were placed in a heating plate where they remained boiling until the volume of nitric acid was reduced in about half. Then, the tubes were cooled and 1mL of perchloric acid ($HClO_4$) was added to each sample, which was reheated until the sample volume reached 2mL, followed by cooling the tubes.

After sample preparation, calcium (Ca), phosphorus (P), potassium (K), sodium (Na), magnesium (Mg), iron (Fe), manganese (Mn), copper (Cu) and zinc (Zn) were determined by

inductively coupled plasma optical emission spectrometry (ICP-OES) (ICPE-9000, Shimadzu do Brasil). The operational conditions are presented in Table 2. In order to ensure the accuracy of the results, Milli-Q water was used to clean the system between each analysis, and after every five samples analyzed at the ICP-OES the system was cleaned with a solution of 1.0% nitric acid.

Table 2. Operational conditions of inductively coupled plasma optical emission spectrometry (ICP-OES) with axial configuration.

Parameter	Technical features
**Radio frequency power (W)**	1200
**Vaporized plasma flow rate (L/min)**	10
**Auxiliary gas flow rate (L/min)**	0.6
**Exposure time (s)**	30
**Nebulizer gas flow rate (L/min)**	0.7
**Nebulizer type**	Concentric
**Spray chamber**	Cyclone
**Number of replicas**	3

The calibration curves were prepared through standardized multi-element solutions of 100mg/L for the elements Ca, Cl, Cu, Fe, K, Mg, Mn, Na, P, and Zn. Calibration curves were prepared from 0.1 to 5mg/L for Cu, Zn, Fe, and Mn, from 0.5 to 100mg/L for Ca, P, Mg, Na and K.

Chloride (Cl) and iodine (I) were not evaluated because the methodology used for digestion in this study does not allow it due to their high ionization energy, for example, iodine, ~~from which~~ only 29.0% of atoms are ionized in argon plasma [7]. Selenium was not evaluated because ~~would need to have~~ a hydride generator coupled to ICP-OES.

The preparation of the wet solution samples, as well as the analyses in ICP-OES, were carried out at the Multi-user Laboratory of Animal Nutrition and Bromatology of the Department of Animal Nutrition and Production of the School of Veterinary Medicine and Animal Science, University of São Paulo, Pirassununga, Brazil.

Determination of the amino acid profile

The amino acid profile was determined by high performance liquid chromatography (HPLC) according to Spitze et al. [8] at the Monogastric Nutrition Laboratory of the Department of Nutrition and Animal Production of the School of Veterinary Medicine and Animal Science, University of São Paulo, Pirassununga, Brazil.

The results were determined in unit per 100 grams of dry matter (DM). It was also calculated how much nutrient each food provides in unit per kg of metabolic weight for animals with low energy requirement, because the FEDIAF (2018) [9] recommendations in unit per 100g of DM takes into account only the equations 95kcal/kg$^{0.75}$ and 110kcal/kg$^{0.75}$ for dogs and 75kcal/kg$^{0.67}$ and 100kcal/kg$^{0.67}$ for cats, however the FEDIAF (2018) [9] reports that there are animals that can present lower metabolizable energy requirement (MER). Therefore, the calculations of unit per kg of metabolic weight were done for dogs with 80 kcal/kg$^{0.75}$ and for cats with 52kcal/kg$^{0.67}$ of MER, equations mentioned in FEDIAF (2018) [9]. For this, the metabolizable energy of the food was estimated by the most current method described in NRC [10].

Statistical analysis

The results obtained were compared with the FEDIAF (2018) [9] recommendations in unit per 100 grams of food and in unit per kg of metabolic weight~~, in a descriptive way~~.

Results

The results of the laboratory analyses of the vegan foods for dogs and the FEDIAF [9] recommendations in unit per 100 grams of dry matter are described in Table 3. All the foods analyzed met the recommendations of the FEDIAF [9] for CP and fat.

Table 3. Nutrient analysis of three commercial vegan diets for dogs and recommended nutrient levels for maintenance of adult dogs, based on a maintenance energy requirement (MER) of 95 kcal of metabolizable energy (ME)/kg$^{0.75}$ (FEDIAF, 2018) - unit per 100 g dry matter.

Nutrient	Foods			FEDIAF 2018	
	**Food A**	**Food B**	**Food C**	**Minimum**	**Maximum**
**Protein**					
**Crude protein (g)**	24.36	27.96	30.04	21.00	NA
**Arginine (g)**	0.77	1.06	0.97	0.60	NA
**Cystine (g)**	0.81	0.95	0.90	NA	NA
**Histidine (g)**	0.54	0.71	0.66	0.27	NA
**Isoleucine (g)**	0.96	1.18	1.24	0.53	NA
**Leucine (g)**	1.53	2.76	3.46	0.95	NA
**Lysine (g)**	1.05	1.35	1.47	0.46	NA
**Methionine (g)**	0.41*	0.71	0.52	0.46	NA
**Methionine + cystine (g)**	1.22	1.66	1.42	0.88	NA
**Phenylalanine (g)**	1.19	1.58	1.74	0.63	NA
**Phenylalanine + tyrosine (g)**	1.99	2.73	3.02	1.03	NA
**Taurine (g)**	0.14	0.16	0.15	NA	NA
**Threonine (g)**	0.90	1.00	1.00	0.60	NA

**Tryptophan (g)**	Not Analysed?	-	-	0.20	NA
**Tyrosine (g)**	0.80	1.14	1.28	NA	NA
**Valine (g)**	1.01	1.19	1.27	0.68	NA
Fat					
**Crude fat (g)**	13.43	13.96	8.08	5.50	NA
**Linoleic acid (ω-6) (g)**	6.57	6.91	3.98	1.53	NA
**Arachidonic acid (ω-6) (mg)**	0	0	0	NA	NA
**Alpha-linolenic acid (ω-3) (g)**	0.93	1.09	0.41	NA	NA
Minerals					
**Ash (g)**	4.91	8.03	8.31	NA	NA
**Calcium (g)**	0.56*	0.86	0.99	0.58	2.50
**Phosphorus (g)**	0.93	1.03	1.03	0.46	1.60
**Ca:P ratio (g)**	0.60*	0.83*	0.96*	1.00	2.00
**Potassium (g)**	0.65	1.27	0.40	0.58	NA
**Sodium (g)**	0.07	0.40	0.35	0.12	NA
**Chloride (g)**	-	-	-	0.17	NA
**Magnesium (g)**	0.19	0.22	0.11	0.08	NA
**Copper (mg)**	3.00**	6.10**	5.30**	0.83	2.80
**Iodine (mg)**	-	-	-	0.12	1.1
**Iron (mg)**	39.00	40.00	75.00	4.17	142.00
**Manganese (mg)**	7.20	5.30	9.60	0.67	17.00
**Selenium (µg)**	-	-	-	35.00	56.8
**Zinc (mg)**	24.00**	18.00	21.00	8.34	22.70

* Less than the minimum recommended by FEDIAF 2018.

**Higher than the maximum recommended by FEDIAF 2018.

NA, not applicable because a minimum or maximum value has not been established for these nutrients.

Compared to the minimum recommendations of FEDIAF [9] for maintenance of adult dogs, one of the vegan foods (Food A) had methionine deficiency. ~~The same Food A also presented calcium deficiency~~. All evaluated foods ~~for dogs~~ ~~presented the~~ Ca:P ratio below the minimum recommendations.

In regard to Cu, all foods evaluated exceeded the maximum safe recommendations for inclusion of this nutrient in dog foods based on FEDIAF [9]. These maximum recommendations are based on the legal limits of the European Union in compliance with the Regulation 1831/2003/EC. Based on these same recommendations, one of the foods evaluated (Food A) also ~~presented~~ excess Zn.

The nutrient analysis of the only commercial vegan food available on Brazilian market formulated for cats and the recommended nutrient levels for maintenance of adult cats by FEDIAF [9] in unit per 100 grams of dry matter are described in Table 4.

Table 4. Nutrient analysis of a commercial vegan food for cats and recommended nutrient levels for maintenance adult cat based on a maintenance energy requirement (MER) of 75 kcal metabolizable energy (ME)/kg$^{0.67}$ (FEDIAF, 2018) - unit per 100 g dry matter.

**Nutrient**	**Food D**	**FEDIAF 2018**	
		**Minimum**	**Maximum**
**Protein**			
**Crude protein (g)**	36.30	33.30	NA
**Arginine (g)**	1.23*	1.3	NA
**Cystine (g)**	1.30	NA	NA
**Histidine (g)**	0.85	0.35	NA
**Isoleucine (g)**	1.48	0.57	NA

**Leucine (g)**	4.05	1.36	NA
**Lysine (g)**	1.65	0.45	NA
**Methionine (g)**	1.03	0.23	NA
**Methionine + cystine (g)**	2.33	0.45	NA
**Phenylalanine (g)**	2.12	0.53	NA
**Phenylalanine + tyrosine (g)**	3.69	2.04	NA
**Taurine (g)**	0.17	0.13	NA
**Threonine (g)**	1.17	0.69	NA
**Tryptophan (g)**	- Not analysed ?	0.17	NA
**Tyrosine (g)**	1.57	NA	NA
**Valine (g)**	1.51	0.68	NA
Fat			
**Crude fat**	17.67	9.00	NA
**Linoleic acid (ω-6)**	8.58	0.67	NA
**Arachidonic acid (ω-6) (mg)**	0*	8.00	NA
**Alpha-linolenic acid (ω-3)**	1.18	NA	NA
Minerals			
**Mineral matter (g)**	6.45	NA	NA
**Calcium (g)**	0.72*	0.79	NA
**Phosphorus (g)**	1.07	0.67	NA
**Ca / P ratio (g)**	0.67*	1.00	2.00
**Potassium (g)**	0.54*	0.80	NA
**Sodium (g)**	0.20	0.10	NA
**Chloride (g)**	-	0.15	NA

**Magnesium (g)**	0.17	0.05	NA
**Copper (mg)**	7.60**	0.67	2.80
**Iodine (mg)**	-	0.17	1.10
**Iron (mg)**	39.00	10.70	142.00
**Manganese (mg)**	7.00	0.67	17.00
**Selenium (µg)**	-	40.00	56.80
**Zinc (mg)**	24.00**	10.00	22.70

* Less than the minimum recommended by FEDIAF 2018.

**Higher than the maximum recommended by FEDIAF 2018.

NA, not applicable because a minimum or maximum value has not been established for these nutrients.

$\frac{1\cdot23}{1\cdot30} \times 100 = 94\cdot6\%$?

Arginine deficiency was observed, for which the food supplied only 59.0% of the minimum recommendations for this nutrient. In addition, the cat food evaluated did not contain any arachidonic acid in its composition, an essential nutrient with a minimum recommendation for cats, according to FEDIAF [9].

The vegan cat food also presented values of calcium, calcium:phosphorus ratio and potassium below the minimum recommendations of FEDIAF [9]. Furthermore, when taking into consideration the maximum recommendations, based on the legal limits of the European Union in compliance with regulation 1831/2003/EC, the vegan food for cats presented levels of copper and zinc above recommendation.

The results expressed in unit per kg of metabolic weight of the vegan dog foods are described in Table 5 and of the vegan cat food in Table 6.

Table 5. Amount of nutrients per kg$^{0.75}$ provided by vegan dog foods with an energy requirement of 80kcal/kg$^{0.75}$ and minimum recommended nutrient levels per kg$^{0.75}$ (FEDIAF, 2018).

Nutrients	Nutrient levels per kg$^{0.75}$			Minimum recommended nutrient levels per kg$^{0.75}$ [10]
	Food A	Food B	Food C	
Protein (g)	5.04	6.11	6.30	4.95
Arginine (g)	0.16	0.23	0.20	0.14
Histidine (g)	0.11	0.16	0.14	0.06
Isoleucine (g)	0.20	0.26	0.26	0.13
Leucine (g)	0.32	0.60	0.73	0.23
Lysine (g)	0.22	0.30	0.31	0.12
Methionine (g)	0.08*	0.16	0.11	0.11
Methionine + cystine (g)	0.25	0.36	0.30	0.21
Phenylalanine (g)	0.25	0.35	0.37	0.15
Phenylalanine + tyrosine (g)	0.41	0.60	0.63	0.24
Threonine (g)	0.19	0.22	0.21	0.14
Tryptophan (g)	NP	NP	NP	0.05
Valine (g)	0.21	0.26	0.27	0.16
Fat	2.79	3.05	1.70	1.51
Linoleic acid (g)	1.36	1.53	0.82	0.36
Minerals				
Calcium (g)	0.12*	0.19	0.21	0.14
Phosphorus (g)	0.19	0.23	0.22	0.11
Potassium (g)	0.13*	0.28	0.08*	0.14

**Sodium (g)**	0.01*	0.09	0.07	0.03
**Chloride (g)**	NP	NP	NP	0.04
**Magnesium (g)**	0.04	0.05	0.02	0.02
**Trace elements**				
**Copper (mg)**	0.63	1.34	1.11	0.20
**Iodine (mg)**	NP	NP	NP	0.03
**Iron (mg)**	8.10	8.77	15.76	1.0
**Manganese (mg)**	1.51	1.17	2.02	0.16
**Selenium (µg)**	NP	NP	NP	8.25
**Zinc (mg)**	4.99	3.86	4.40	2.0

* Less than the minimum recommended by FEDIAF 2018.

NP, not performed; ND, not detected. — what is this?

previous tables as well then

Table 6. Amount of nutrients per kg$^{0.67}$ provided by a vegan cat food with an energy requirement of 52 kcal/kg$^{0.67}$ and minimum recommended nutrient levels per kg$^{0.67}$ (FEDIAF, 2018).

**Nutrients**	**Nutrient levels per kg$^{0.67}$**	**Minimum recommended**
	**Food D**	**nutrient levels per kg$^{0.67}$ [10]**
**Protein (g)**	4.71*	6.25
**Arginine (g)**	0.16*	0.25
**Histidine (g)**	0.11	0.08
**Isoleucine (g)**	0.19	0.12
**Leucine (g)**	0.53	0.29
**Lysine (g)**	0.21	0.09

**Methionine (g)**	0.13	0.04
**Methionine + cystine (g)**	0.3	0.09
**Phenylalanine (g)**	0.27	0.12
**Phenylalanine + tyrosine (g)**	0.48	0.44
**Taurine (g)**	0.02*	0.15
**Threonine (g)**	0.15	0.04
**Tryptophan (g)**	NP	0.15
**Valine (g)**	0.2	0.03
**Fat**	2.29	2.25
**Linoleic acid (g)**	1.11	0.13
**Arachidonic acid (mg)**	ND*	1.50
**Minerals**		
**Calcium (g)**	0.09*	0.15
**Phosphorus (g)**	0.14	0.13
**Potassium (g)**	0.07*	0.15
**Sodium (g)**	0.02	0.02
**Chloride (g)**	NP	0.03
**Magnesium (g)**	0.02	0.01
**Trace elements**		
**Copper (mg)**	0.98	0.13
**Iodine (mg)**	NP	0.03
**Iron (mg)**	5.07	2.00
**Manganese (mg)**	0.91	0.13
**Selenium (μg)**	NP	7.50

**Zinc (mg)**	3.11	1.88

* Less than the minimum recommended by FEDIAF 2018.

NP, not performed; ND, not detected.

Regarding the recommendations of nutrients per kg of metabolic weight for animals with lower MER, the same deficiencies were observed when comparing the results in unit per 100 grams of dry matter. Beside these deficiencies, values below the minimum recommendation for protein and taurine in cat food were observed.

Discussion

Laboratory analyses of these four vegan foods showed that none of them met all of the nutritional recommendations of FEDIAF [9] for the species for which they were intended. Levels below the minimum recommendation for various nutrients were observed, including amino acids such as arginine and methionine, and minerals such as calcium, potassium and sodium. The main findings of this study were levels below the detection limit for arachidonic acid (essential for cats), as well as failure to comply with the minimum recommendation for the Ca:P ratio in all analyzed foods.

Arachidonic acid (AA) is an essential fatty acid for cats due to the low activity of the enzyme delta-6-desaturase in this species, which is responsible for the conversion of linoleic acid into AA. In general, fats of animal origin are good sources of AA, whereas vegetable oils do not ~~present~~ contain this fatty acid ~~in its composition~~. The study of lipid metabolism in felines instigated the interest of the scientific community in the 1970s when Rivers et al. [11] observed while studying liver samples that cats had very low activity of the delta-6-dessaturase enzyme. After this discovery, effects of low AA diets for cats were studied and it has been shown that

AA deficiency in cats can result in thrombocytopenia, impairment of platelet aggregation and inability to conceive [12]. Pawlosky and Salem [13] tested AA-free diets supplemented with 1% corn oil for cats before mating and throughout gestation. All females cycled and matched, but a high incidence of congenital defects and low litter viability was observed. The vegan foods analyzed in our study used soybean oil as the only source of fat, so it was expected that AA would not be detected in these products. Some marine algae such as *Ascophyllum nodosum* may have AA in their composition and could, therefore, be used as a source of this fatty acid. On the label of the commercial vegan cat food analyzed it was stated that there was an inclusion of algae flour, although the information about which species of algae and the amount added were not included.

All foods presented a Ca:P ratio below the minimum recommended by FEDIAF [9] of 1:1. Regarding Ca, two foods (one for dogs and one for cats) presented concentrations below recommendations. A similar result was found by Gray et al. [14], who analyzed two commercial vegan foods for cats and one of them presented calcium concentrations well below the minimum recommendation for the species. In general, by-products of animal origin have higher calcium concentrations when compared to products of plant origin [9], which may justify the results found. However, according to label information, there was an inclusion of calcium supplementation in the form of calcium carbonate and dicalcium phosphate in the four analyzed foods. Even so, the values found were below the minimum recommendation. In humans, it has been shown that vegan people consume less calcium [15] and present lower bone mineral density [16,17] compared to non-vegans, which may increase the risk of bone fracture [18].

The Ca:P ratio below the minimum recommendation and the low concentrations of Ca in the diet may result in secondary nutritional hyperparathyroidism (SNH) in dogs and cats [19, 20]. This alteration was quite common in the past because of the unbalanced feeding that was

provided to pets [20, 21, 22, 23, 24] However, nowadays SNH is uncommon due to the large use of complete and balanced commercial foods that supply the animal's calcium requirements. The SNH may result in rubber jaw syndrome, a group of lesions characterized by the loss of the lamina dura of the teeth, loose teeth, and osteopenia of the skull bones, and this rubber aspect is consequent to the replacement of the mineral structure by fibrous tissue [24]. Furthermore, hypocalcemia is usually present in this syndrome, which can cause muscle spasms, convulsion, and osteopenia, which causes bone fractures [20].

One of the dog foods did not meet the minimum recommendation for the amino acid methionine. This result was expected since methionine ~~is in small concentrations~~ in vegetable protein based diets [10]. It was stated on the product label that DL-methionine was included, but even so, the concentrations were below the minimum recommendation. Currently, there is a concern regarding methionine for dogs since the low consumption of this amino acid may be indirectly related to an increased risk of developing dilated cardiomyopathy (DC) [25]. Methionine is a precursor of taurine, an important amino acid for myocardial function [26, 27]. Dogs are able to synthesize taurine from sulfur amino acids such as methionine and cysteine, mainly in the liver and central nervous system, through the transsulfuration pathway [26]. The relationship between taurine deficiency and DC in dogs began to be studied by Kramer et al. [28], who observed that a subset of dogs with DC had taurine deficiency. Further studies have shown that taurine supplementation for dogs with DC associated with the deficiency of this amino acid resulted in resolution of myocardial changes and allowed the removal of support medication for most of these dogs [27, 29, 30, 31]. These data suggest a direct link between taurine deficiency and DC development. Fascetti et al. [27] assessed three taurine-deficient Golden Retrievers that were fed a vegetarian diet formulated by the owner, according to the authors, this deficiency may have occurred due to possible diet deficiency in sulfur amino acids precursors of taurine.

This taurine deficiency may occur as a consequence of dietary deficiency of methionine or cysteine, however other nutritional factors may be involved, such as decreased bioavailability of the sulfur amino acids that are precursors of taurine, abnormal enterohepatic bile acid recycling, altered taurine intestinal metabolism and urinary loss of taurine [25, 32].

The vegan food analyzed which presented a low concentration of methionine may represent a risk factor for the development of DC. In addition, this food presented a high inclusion of soybean meal in its composition, which was the main protein ingredient used in the formulation of this food. It has been observed in cats that soy protein decreases plasma taurine concentrations as a consequence of increased bile acid loss that is conjugated to taurine through microbial degradation and acceleration of cholecystokinin-mediated bile acid turnover [33], thus the high inclusion of this ingredient may also possibly contribute to the development of this disease.

The analyzed cat food did not meet the minimum recommendation of arginine for this species. This result corroborates a previous study ~~developed~~ by Gray et al. [14]. This result was expected because arginine is usually found in small amounts in plant-based diets and according to the product label, the food was not supplemented with arginine. Cats are more sensitive to arginine deficiency than dogs [9, 34]. Arginine acts as a precursor of ornithine (intermediate) in the urea cycle. This allows large quantities of ammonia from a high-protein meal to be converted to urea for further excretion [35]. Lack of arginine in cats may lead to hyperammonemia and severe uremia causing symptoms such as emesis, ataxia, muscle spasms, and hyperesthesia. [36]. The higher sensitivity of cats to arginine-deficient diets may be explained by two reasons. Most animals are able to use glutamate and proline as precursors for the synthesis of ornithine in the intestinal mucosa but cats are unable to utilize these amino acids as precursors because of the low concentration of active pyrroline-5-carboxylate synthetase enzyme in their intestinal mucosa [37]. In addition, cats present low activity of

another essential enzyme for this process, the ornithine aminotransferase [35]. Furthermore, felines are unable to synthesize arginine from ornithine, even if they consume a diet with high amounts of ornithine. It occurs due to the fact that cats are unable to synthesize citrulline in the intestinal mucosa, a molecule that acts as a precursor in the synthesis of arginine from ornithine. Although they produce citrulline in the liver, this molecule is unable to exit the hepatocytes and be transported to the kidneys, where conversion to arginine could potentially occur [38].

In addition to the nutritional recommendation based on DM, FEDIAF [9] also has recommendations per kg of metabolic weight for dogs and cats. This is important since recommendations based on DM for cats take into account ~~the~~ MER of 75kcal/kg$^{0.67}$ and 100kcal/kg$^{0.67}$, although cats may present lower MER. FEDIAF [9] suggests that MER of inactive adult cats can range from 52 to 75kcal per kg of metabolic weight. Loureiro et al. [39] reported mean MER for adult cats of 59.5±2.2 Kcal/BW$^{0.67}$/day. For dogs, this comparison is also important, since nutritional recommendations based on DM take into account the energy requirement of 95kcal/kg$^{0.75}$ and 110kcal/kg$^{0.75}$, but there are animals with lower energy requirements. According to the recommendations of the FEDIAF [9], the MER of inactive dogs can vary from 80 to 120kcal/kg$^{0.75}$. Pedrinelli et al. [40] observed mean MER for adult dogs of 86.1±19.8 kcal/BW$^{0.75}$. For those animals with low MER, the nutrient concentrations in the food should be higher, to ensure the intake of the minimum ~~recommendation~~. Therefore, a comparison was made with recommendations per kg of metabolic weight for cats with MER of 52kcal/kg$^{0.67}$, and dogs with MER of 80kcal/kg$^{0.75}$ as mentioned by FEDIAF [9].

The results per kg of metabolic weight showed, besides the same deficiencies found in the results per unit of DM, values of protein and taurine below the minimum recommendation in the cat food. These data corroborate with a study ~~developed~~ by Loureiro et al. [39] in which it was observed that a commercial food with 28% CP in DM basis did not meet the minimum recommendation of protein intake in g/kg$^{0.67}$, although it did support the requirement for CP based

on DM. This lack of protein intake was due to the low amount of food ingested by these animals to maintain weight, which had an average MER of 59.5 kcal/kg$^{0.67}$. In the study, the cats that consumed this diet during a 60-day period lost 90 grams of lean body mass. It was also observed that the protein intake required to maintain stable lean body mass was 5.17g/kg$^{0.67}$, higher than that provided by the vegan food analyzed in our study for cats. Based on this previous study, it is possible that the intake of the vegan cat food evaluated may lead to risk of lean body mass loss in cats with low energy requirements, despite presenting 36.3% of CP in DM.

Regarding taurine consumption below the recommendation per kg$^{0.67}$, two previous studies have also observed a deficiency of this amino acid in commercial vegetarian and vegan cat foods [14, 41]. Regarding the implications of cats consuming a plant-based diet, Wakefield et al. [42] evaluated the blood concentrations of taurine in cats that consumed vegetarian diets and observed that, of the 17 cats evaluated, three had taurine concentrations below the reference range.

Taurine is an amino acid only found in animal products. In cat foods, even with the inclusion of animal ingredients, taurine supplementation is usually required to achieve the minimum ~~recommendation~~ for this amino acid. In the case of diets without animal products, taurine supplementation becomes mandatory. The vegan cat food analyzed in this study had taurine supplementation, however, the concentration found was below the recommendation, based on unit per kg$^{0.67}$ for cats with MER of 52kcal/kg$^{0.67}$.

Taurine is an essential amino acid for cats. This species conjugates bile acids exclusively through taurine, unlike dogs which may occasionally carry out this conjugation with glycine [14, 43]. The intestinal lumen microbial degradation increases the loss of taurine in cats [44]. In addition, cats show limited hepatic production of taurine from sulfur amino acids, such as methionine and cysteine, resulting from the low activity of the enzyme cysteine dioxygenase. This low enzymatic activity limits the production of cysteine sulfate, an

intermediate in taurine synthesis. Felines also have limited activity of the enzyme cysteine sulfinic decarboxylase, responsible for converting cysteine sulfate to taurine and have a high activity of the enzyme aspartate aminotransferase, which transforms cysteine sulfate into pyruvate, rather than allowing it to be decarboxylated for hypotaurine and therefore into taurine [44, 45, 46]. All these associated factors result in the dietary requirement for taurine in felines.

It is important to emphasize that this vegan food analyzed may be a risk factor for taurine deficiency due to the high inclusion of soybean meal in its composition. It is known that soy protein may decrease plasma concentrations of taurine in cats when compared to casein, which seems to be related to increased use of taurine for conjugation of bile acids, increased intestinal loss of taurine by microbial degradation and accelerated cholecystokinin-mediated [33, 47, 48]. Therefore, caution with supplementation of taurine in vegan foods with high inclusion of soy protein should be even higher.

In cats, dietary deficiency of taurine may lead to cardiac abnormalities such as dilated cardiomyopathy, since taurine is related to myocardial function and it is essential for the maintenance of cardiac contractility [49,50]. In a study developed by Novotny et al. [51], a diet deficient in taurine was provided for 23 cats over a period of 6 to 15 months and most of the animals presented decreased myocardial function assessed by echocardiography.

Taurine also plays an important role in maintaining the structural integrity of the retina and dietary deficiency of this amino acid can lead to photoreceptor cell degeneration [52, 53, 54, 55, 56]. In a study carried out by Jacobson et al. [56], eight male cats were fed a diet deficient in taurine for 5 to 9 months. All animals developed abnormalities in the retina detected by fundoscopy and electroretinography. In addition, taurine deficiency can impair the reproductive performance of cats with consequences on ~~the~~ development ~~of pups~~ [57].

Vitamin analyses were not performed in the present study, however, the label of the vegan cat food analyzed reported vitamin D inclusion in the form of vitamin D_2

(ergocalciferol), which is present in vegetables. It is known that in cats, vitamin D_2 is not as efficient at maintaining 25(OH)D serum concentrations as vitamin D_3 (cholecalciferol) [58]. The 25(OH)D is intermediate metabolite of vitamin D. It is synthesized in the liver and converted to $1,25(OH)_2D$ in the proximal renal tubules [59]. $1,25(OH)_2D$ is the active metabolite that performs classical hormonal functions related to bone metabolism and calcium and phosphorus homeostasis in the body, besides non-classical functions such as immune-modulating activity [60,61,62], protective activity against autoimmune reaction [63, 64], antitumoral activity [65,66,67,68] and anti-inflammatory activity [69,70]. Because of this particularity of cats in relation to vitamin D metabolism, if the analyzed vegan food used concentrations close to the minimum recommendation this may result in long-term vitamin D deficiency, because although vitamin D was not quantified in the present study, the food label demonstrates that the most suitable vitamin D source for cats was not used.

High concentrations of copper and zinc were found in the diets evaluated. There is insufficient information in the literature about the safe upper limit for copper and zinc intake for dogs and cats, so it is not known if levels observed in these foods may imply health risks for dogs and cats.

This study had as limitations the non-analysis of other essential nutrients, such as water-soluble and fat-soluble vitamins, besides the iodine, chloride and selenium minerals.

In conclusion, all foods had one or more nutrients lower than the recommendations and some presented zinc and copper excess. Therefore, these foods should not be recommended for dogs and cats, although they are available in the Brazilian market, because dietary deficiencies observed may lead to health risks for dogs and cats. Furthermore, manufacturers should review their formulations to ensure the nutritional adequacy of these foods.

Acknowledgments

We thank Renata Maria Consentino Conti and Simi Luisa Durante Aflalo for technical assistance.

Author Contributions

R.V.A.Z.; T.H.A.V. and M.A.B. were responsible for planning the study. R.V.A.Z.; L.W.R.; T.H.A.V.; R.B.A.R.; F.A.T.; M.F.R. and V.P. were responsible conducting the analysis, writing and editing the manuscript. M.A.B were responsible for reviewing the manuscript.

References

1. Dodd AS, Cave NJ, Adolphe JL, et al. Plant-based (vegan) diets for pets: A survey of pet owner attitudes and feeding practices. PloS one 2019;14(1), e0210806.
2. IBOPE INTELIGÊNCIA – pesquisa de opinião pública sobre o vegetarianismo-JOB0416/2018 BRASIL.
3. Kienzle E, & Engelhard RA. Field study on the nutrition of vegetarian dogs and cats in Europe. Compendium on Continuing Education for the Practicing Veterinarian 2001; 23(9), 81-81.
4. AOAC. Official methods of analysis. 2006(AOAC International, 2006).
5. Williams, R. D. & Olmsted, W. H. A biochemical method for determining indigestible residue (crude fiber) in feces: lignin, cellulose, and non-water soluble hemicelluloses. J. Biol. Chem. 1935; 108, 635–666.
6. Animal, I. S. O., and Vegetable Fats. "Oils-Preparation of Methyl Esters of Fatty Acids (method ISO 5509)." Geneva: International Organization for Standardization (1978).

7. Montaser A. Inductively coupled plasma mass spectrometry. Washington D.C., EUA: Wiley-VCH, 1998.

8. Spitze AR, Wong DL, Rogers QR, et al. Taurine concentrations in animal feed ingredients; cooking influences taurine content. J Anim Physiol Anim Nutr (Berl) 2003; 87:251–262.

9. FEDIAF. Nutritional Guidelines for Complete and Complementary Pet Food for Cats and Dogs, 2018. (Fédération Européenne de l'Industrie des Aliments pour Animaux Familiers, 2018).

10. National Research Council. Nutrient requirements of dogs and cats. National Academies Press, 2006.

11. Rivers JPW, Sinclair AJ & Crawford MA. Inability of the cat to desaturate essential fatty acids. Nature 1875; 258(5531), 171.

12. MacDonald ML, Anderson BC, Rogers QR, et al. Essential fatty acid requirements of cats: pathology of essential fatty acid deficiency. American journal of veterinary research 1984; 45(7), 1310-1317.

13. Pawlosky RJ, & Salem Jr N. Is dietary arachidonic acid necessary for feline reproduction. The Journal of nutrition 1996; 126(suppl_4), 1081S-1085S.

14. Gray CM, Sellon RK & Freeman LM. Nutritional adequacy of two vegan diets for cats. Journal of the American Veterinary Medical Association 2004; 225(11), 1670-1675.

15. Davey GK, Spencer EA, Appleby PN et al. EPIC–Oxford: lifestyle characteristics and nutrient intakes in a cohort of 33 883 meat-eaters and 31 546 non meat-eaters in the UK. Public health nutrition 2003; 6(3), 259-268.

16. Chiu JF, Lan SJ, Yang CY, et al. Long-term vegetarian diet and bone mineral density in postmenopausal Taiwanese women. Calcif Tissue Int 1997; 60:245–9.

17. Lau EMC, Kwok T, Woo J, Ho SC. Bone mineral density in Chinese elderly female vegetarians, vegans, lacto-vegetarians and omnivores. Eur J Clin Nutr 1998; 52:60–4.

18. Craig WJ. Health effects of vegan diets. The American journal of clinical nutrition 2009; 89(5), 1627S-1633S.

19. NAP RC & HAZEWINKEHL AW. Growth and skeletal development in the dog in relation to nutrition; a review. Veterinary Quarterly 1994; 1. 50-59.

20. Tomsa K, Glaus T, Hauser et al. Nutritional secondary hyperparathyroidism in six cats. Journal of small animal practice 1999; 40(11), 533-539.

21. Svanberg G, Lindhe J, Hugoson A et al. Effect of nutritional hyperparathyroidism on experimental periodontitis in the dog. European Journal of Oral Sciences 1973; 81(2), 155-162.

22. Cook SD, Skinner HB, Haddad RJ. A quantitative histologic study of osteoporosis produced by nutritional secondary hyperparathyroidism in dogs. Clin Orthop 1983; 175:105-120.

23. Kawaguchi K, Braga III IS, Takahashi A, et al. Nutritional secondary hyperparathyroidism occurring in a strain of German shepherd puppies. Japanese Journal of Veterinary Research 1993; 41(2-4), 89-96.

24. Fornel-Thibaud P, Blanchard G, Escoffier-Chateau L et al. Unusual case of osteopenia associated with nutritional calcium and vitamin D deficiency in an adult dog. Journal of the American Animal Hospital Association 2007; 43(1), 52-60.

25. Freeman LM, Stern JA, Fries R et al. Diet-associated dilated cardiomyopathy in dogs: what do we know?. Journal of the American Veterinary Medical Association 2018; 253(11), 1390-1394.

26. Hayes KC. Taurine nutrition. Nutr Res Rev 1988; 1:99–113.

27. Fascetti AJ, Reed JR, Rogers QR et al. Taurine deficiency in dogs with dilated cardiomyopathy: 12 cases (1997–2001). Journal of the American Veterinary Medical Association 2003; 223(8), 1137-1141.

28. Kramer GA, Kittleson MD, Fox PR et al. Plasma taurine concentrations in normal dogs and in dogs with heart disease. Journal of veterinary internal medicine 1995; 9(4), 253-258.

29. Bélanger MC, Ouellet M, Queney G et al. Taurine-deficient dilated cardiomyopathy in a family of golden retrievers. Journal of the American Animal Hospital Association 2005; 41(5), 284-291.

30. Kittleson MD, Keene B, Pion PD et al. Results of the multicenter spaniel trial (MUST): taurine- and carnitine- responsive dilated cardiomyopathy in American cocker spaniels with decreased plasma taurine concentration. Journal of Veterinary Internal Medicine 1997; 11(4), 204-211.

31. Backus RC et al. Taurine deficiency in Newfoundlands fed commercially available complete and balanced diets. Journal of the American Veterinary Medical Association 2003; 223.8: 1130-1136.

32. Ko KS, Fascetti AJ. Dietary beet pulp decreases taurine status in dogs fed low protein diet. J Anim Sci Technol 2016; 58:29.

33. Kim SW, Morris JG, Rogers QR. Dietary soybean protein decreases plasma taurine in cats. J Nutr 1995; 125:2831–2837.

34. Czarnecki GL and Baker DH. Urea cycle function in the dog with emphasis on the role of arginine. J. Nutr. 1984; 114:581-590.

35. Morris JG. Nutritional and metabolic responses to arginine in carnivores. J. utr. 1985; 115:524-531.

36. Morris JG & Rogers QR. Ammonia intoxication in the near-adult cat as a result of a dietary deficiency of arginine. Science 1978; 199(4327), 431-432.

37. Rogers R. and Phang JM. Deficiency of pyrroline-5-carboxylale synthase in the intestinal mucosa of the cat. J. Nutr. 1985; 115: 146-150.

38. Kirk CA, Debraekeleer J, Armstrong PJ. Normal cats. In: Hand MS, Thatcher CD, Remillard RL, et al, eds. Small animal clinical nutrition. 4th ed. Philadelphia: WB Saunders Co, 2000; 291–347.

39. Loureiro BA, Garcia CA, Peres FM et al. Markers of protein metabolism in adult cats fed diets with different protein and starch contentes. in Proceedings. 22nd Congress of the European Society Of Veterinary and Comparative Nutrition 2018; 123-123.

40. Pedrinelli V, Porsani MYH, Lima DM et al. Predictive equations of maintenance energy requirement of healthy and chronically ill adult dogs. in Proceedings. 22nd Congress of the European Society Of Veterinary and Comparative Nutrition 2018; 236-236.

41. Kanakubo K, Fascetti AJ & Larsen JA. Assessment of protein and amino acid concentrations and labeling adequacy of commercial vegetarian diets formulated for dogs and cats. Journal of the American Veterinary Medical Association 2015; 247(4), 385-392.

42. Wakefield LA, Shofer FS, & Michel KE. (2006). Evaluation of cats fed vegetarian diets and attitudes of their caregivers. Journal of the American Veterinary Medical Association 2006; 229(1), 70-73.

43. Rentschler LA, Hirschberger LL & Stipanuk MH. Response of the kitten to dietary taurine depletion: effects on renal reabsorption, bile acid conjugation and activities of enzymes involved in taurine synthesis. Comparative biochemistry and physiology. B, Comparative biochemistry 1986; 84(3), 319-325.

44. Morris JG, Rogers QR, Kim SW, et al. Dietary taurine requirement of cats is determined by microbial degradation of taurine in the gut. Vet Clin Nutr 1994; 1:118–127.

45. Morris JH and Rogers QR. The metabolic basis for the taurine requirement of cats. Taurine 1992. Springer, Boston, MA. 33-44.

46. Pion PD, Sanderson SL, Kittleson MD. The effectiveness of taurine and levocarnitine in dogs with heart disease. Vet Clin North Am Small Anim Pract 1998; 28:1495–1514.

47. Hickman MA, Bruss ML, Morris JG, et al. Dietary protein source (soybean vs. casein) and taurine status affect kinetics of the enterohepatic circulation of taurocholic acid in cats. J Nutr 1992; 122:1019–1028.

48. Backus RC, Rogers QR, Rosenquist GL, et al. Diets causing taurine depletion in cats substantially elevate postprandial plasma cholecystokinin (CCK) concentration. J Nutr 1995; 125:2650–2657.

49. Schaffer SW, Jong CJ, Ramila KC et al. Physiological roles of taurine in heart and muscle. Journal of Biomedical Science 2010; 17(1), S2.

50. Schaffer SW, Shimada-Takaura K, Jong CJ et al. Impaired energy metabolism of the taurine-deficient heart. Amino Acids 2016; 48(2), 549-558.

51. Novotny MJ, Hogan PM & Flannigan G. Echocardiographic evidence for myocardial failure induced by taurine deficiency in domestic cats. Canadian Journal of Veterinary Research 1994; 58(1), 6.

52. Hayes KC, Carey RE and Schmidt SY. Retinal degeneration associated with taurine deficiency in the cat. Science 1975; 188.4191: 949-951.

53. Barnett KC and Burger IH. Taurine deficiency retinopathy in the cat. Journal of Small Animal Practice 1980; 21.10: 521-534.

54. Hayes KC. Nutritional problems in cats: taurine deficiency and vitamin A excess. The Canadian Veterinary Journal 1982; 23.1: 2.

55. Imaki H, Moretz RC, Wisniewski HM et al. Feline maternal taurine deficiency: effects on retina and tapetum of the offspring. Developmental neuroscience 1986; 8(3), 160-181.

56. Jacobson SG, Kemp CM, Borruat FX et al. Rhodopsin topography and rod-mediated function in cats with the retinal degeneration of taurine deficiency. Experimental eye research 1987; 45(4), 481-490.

57. Sturman JÁ and Jeffrey MM. High dietary taurine effects on feline tissue taurine concentrations and reproductive performance. The Journal of nutrition 1992; 122.1: 82-88.

58. Morris JG. Cats discriminate between cholecalciferol and ergocalciferol. Journal of animal physiology and animal nutrition 2002; 86:229-238.

59. Brito Galvao JF, Nagode LA, Schenck PA et al. Calcitriol, calcidiol, parathyroid hormone, and fibroblast growth factor- 23 interactions in chronic kidney disease. Journal of Veterinary Emergency and Critical Care 2013; 23(2), 134-162.

60. Liu PT, Stenger S, Li H et al. Toll-like receptor triggering of a vitamin D-mediated human antimicrobial response. Science 2006; 3111:770-1773.

61. Motlagh BM, Ahangaran NA & Froushani SMA. Calcitriol modulates the effects of bone marrow-derived mesenchymal stem cells on macrophage functions. Iranian journal of basic medical sciences 2015; 18(7), 672.

62. Rodriguez-Cortes A, Martori C, Martinez-Florez A et al. Canine Leishmaniasis Progression is Associated with Vitamin D Deficiency. Scientific reports 2017; 7:3346.

63. Pludowski P, Holick MF, Pilz S et al. Vitamin D effects on musculoskeletal health, immunity, autoimmunity, cardiovascular disease, cancer, fertility, pregnancy, dementia and mortality—a review of recent evidence. Autoimmunity reviews 2013; 12:976-989.

64. Laws EJ, Kathrani A, Harcourt-Brown TR et al. 25- Hydroxy vitamin D3 serum concentration in dogs with acute polyradiculoneuritis compared to matched controls. Journal of Small Animal Practice 2018; 59:222-227.

65. Kunakornsawat S, Rosol TJ, Capen CC et al. Effects of 1, 25 (OH) 2D3, EB1089, and analog V on PTHrP production, PTHrP mRNA expression and cell growth in SCC 2/88. Anticancer research 2001; 21(5), 3355-3363.

66. Kunakornsawat S, Rosol TJ, Capen CC et al. Effects of 1, 25 (OH) 2D3, 25OHD3, and EB1089 on cell growth and Vitamin D receptor mRNA and 1α-hydroxylase mRNA expression in primary cultures of the canine prostate. The Journal of steroid biochemistry and molecular biology 2004; 89, 409-412.

67. Rassnick KM, Muindi JP, Johnson CS et al. In vitro and in vivo evaluation of combined calcitriol and cisplatin in dogs with spontaneously occurring tumors. Cancer chemotherapy and pharmacology 2008; 62:881-891.

68. Malone EK, Rassnick KM, Wakshlag JJ et al. Calcitriol (1, 25-dihydroxycholecalciferol) enhances mast cell tumor chemotherapy and receptor tyrosine kinase inhibitor activity in vitro and has single- agent activity against spontaneously occurring canine mast cell tumors. Veterinary and comparative oncology 2010; 8:209-220.

69. Jaffey J, Amorim J & DeClue AE. In vitro effects of vitamin D on phagocytosis, TLR4, and cytokine production. J Vet Intern Med 2017; 31(4), 775.

70. Jaffey JÁ, Amorim J, Declue AE. Effect of calcitriol on in vitro whole blood cytokine production in critically ill dogs. The Veterinary Journal 2018, v. 236, p. 31-36.
